# Advancements in Functional Dressings and a Case for Cotton Fiber Technology: Protease Modulation, Hydrogen Peroxide Generation, and ESKAPE Pathogen Antibacterial Activity

**DOI:** 10.3390/ijms27020610

**Published:** 2026-01-07

**Authors:** J. Vincent Edwards, Nicolette T. Prevost, Doug J. Hinchliffe, Sunghyun Nam, Crista A. Madison

**Affiliations:** Southern Regional Research Center, Agricultural Research Service, United States Department of Agriculture, New Orleans, LA 70124, USA; nicolette.prevost@usda.gov (N.T.P.); doug.hinchliffe@usda.gov (D.J.H.); sunghyun.nam@usda.gov (S.N.); crista.madison@usda.gov (C.A.M.)

**Keywords:** wound dressings, wound healing, antibacterial, ESKAPE model

## Abstract

The development of functionality in wound dressings has progressed since the discovery by Winter that moist wounds heal more rapidly. Approaches to incorporate functionality on several fronts of wound healing have been targeted. Here, we consider three functional features that have received increased attention for their role in promoting healing in hard-to-heal wounds: control of protease levels, hydrogen peroxide generation, and antibacterial efficacy against multidrug resistance bacteria, the ESKAPE (*Enterococcus faecium, Staphylococcus aureus*, *Klebsiella pneumoniae*, *Acinetobacter baumannii*, *Pseudomonas aeruginosa*, and *Enterobacter species*) pathogens. We review some clinically employed dressings used to treat chronic and burn wounds that have been characterized by their functional protease-modulating activity and contrast one well-studied analog with a cotton-based technology. Similarly, hydrogen peroxide generation profiles were obtained for dressings in different moist wound healing categories and contrasted with a modified form of a known hemostatic cotton-based technology. We examined ascorbic acid-modified forms of a cotton-based technology used for bleeding control in an ESKAPE antibacterial assessment using the AATCC 100 TM. The results for the cotton-based technology were significant protease uptake, hydrogen peroxide generation capacities at proliferative and antimicrobial levels, and >99.99% efficacy against ESKAPE pathogens. These results reflect the importance of considering new forms of cotton fiber technology for incorporation in advanced wound dressing approaches.

## 1. Introduction

### 1.1. Toward Intelligent Dressing Treatments

The process of wound healing may be divided into four distinct but overlapping phases: (1) hemostasis, (2) inflammation, (3) proliferation, and (4) remodeling. There are both physical and molecular markers that have been identified as key to skin repair as part of each of these phases to advance a robust trajectory to healing. Among the physical properties, moisture control, exudate composition and volume, and temperature are often cited as measurable markers that influence healing. Molecular markers in the literature were reviewed by Linsey Lindley and others around 2016 [1], and the importance of understanding mechanisms in aberrant wound healing was addressed for monitoring and predicting wound healing outcomes. However, nine years later at this time few of the molecular markers cited, except for proteases, had undergone widespread adoption in diagnostic and treatment applications. The molecular markers that have been of benefit include pH, proteases, growth factors, and reactive oxygen species, which have been studied to some extent with attempts to clinically modulate or deliver restorative function through modified dressings and topical formularies.

### 1.2. The Inflammatory Phase and Protease Burden

Inflammation is a defense response of the immune system that occurs upon disruption of tissue homeostasis [2]. However, in prolonged disease, the inflammatory response upregulates neutrophils and macrophages [3], which release pathological levels of proteases, cytokines and lipids; hence, a systemic amplification of inflammatory molecules and degradative enzymes promotes chronic inflammation. Though progress continues to be made, the complex problems associated with chronic wound treatment and prevention remain as challenging both medically and economically as any other major inflammatory disease [4,5].

**Figure 1:**
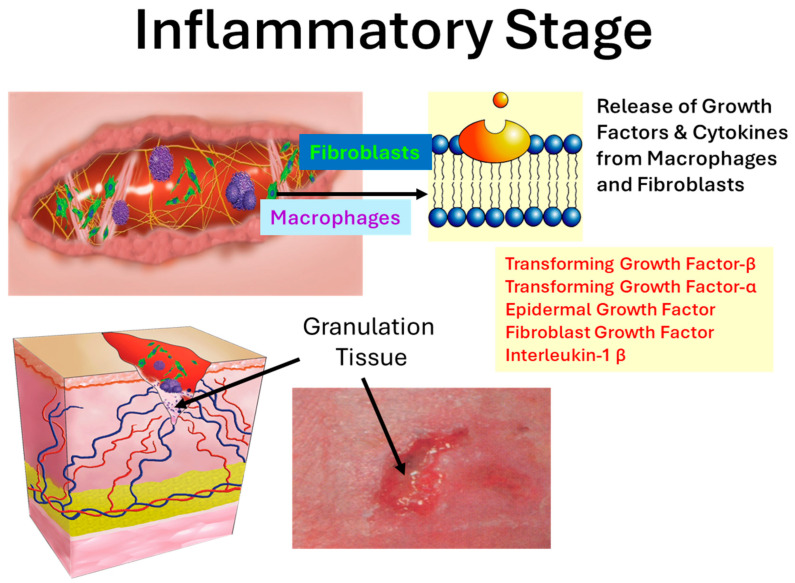
The inflammatory and proliferative phase of wound healing growth factors enhance proliferation of fibroblasts, macrophages, and keratinocytes. By days 5 through 7, the fibroblasts have started to lay down new collagen and glycosaminoglycans, which form the core of the wound that is stabilized in granulation tissue. However, wound healing does not always occur in a predictable fashion. A chronic wound is one that fails to heal in a timely manner due to pathological conditions. The prolonged inflammatory phase that occurs in non-healing wounds is often marked by a massive ingestion of neutrophils that have a high titer of proteolytic enzymes. In a healing wound, neutrophils facilitate antibacterial, host defense and debridement as depicted here. (Figure is not drawn to scale, illustrative only).

Wound healing does not always occur in a predictable fashion. Numerous local and systemic factors may influence the pattern and rate of healing, and can lead to a chronic, non-healing state. A chronic wound is one that fails to heal in a timely fashion due to one or more pathologies precipitated by the onset of conditions, including infection, radiation damage, hypoxia, diabetes, venous insufficiency, malnutrition or ingestion of pharmacological agents. The result is a stalled healing process trapped in the inflammatory stage that inhibits. However, as depicted in Figure 1, restoration of a normal inflammatory phase improves the trajectory to wound healing.

Notably, in a healing wound, the interaction of growth factors, the extracellular matrix (ECM), and cells are central to the process of wound healing. A variety of growth factors and cytokines, including fibroblast growth factor (FGF), transforming growth factor (TGF), transforming growth factor (EGF), interleukin-1 beta (IL-1β), interleukin- 1 alpha (IL-1 α), and tumor necrosis factor (TNF), are involved with the ECM to activate cell proliferation. Relevant to the study at hand is that some of these participate in protease modulation of matrix metalloproteases (MMP) and tissue inhibitors of metalloproteinases (TIMP). For example, it has been shown that TNF alpha plays a role in the synthesis of collagen, MMP and TIMP, and acts to modulate ECM degradation through modulation of these. IL-1 alpha also associated with proinflammatory activity is increased in chronic wounds and decreases in acute wounds as is collagenase [6,7]. MMPs play a pivotal role in normal wound healing via the degradation of ECM components, which facilitate the migration of cells and remodeling of the wound. Degradation and remodeling of the ECM by proteases is part of the complex series of events termed dynamic reciprocity characterized by reciprocal interaction of ECM growth factors and cells [8]. MMPs play a role in both releasing growth factors from the ECM and clearing ECM proteins to expose areas that facilitate activation of growth factor receptors. MMP degradation of ECM also influences leukocyte influx, angiogenesis, and tissue remodeling. For example, matrikines, which are signaling peptides with similar amino acid sequences, have lower receptor binding affinity than their analogous growth factor proteins and are embedded subcomponents of the ECM released by way of proteolysis with MMP. Tenascin and laminin are examples of matrikines that stimulate activation of FGF receptors yielding fibroblast and keratinocyte release. Moreover, tenascin C and laminin-332 are expressed by keratinocytes at the leading edge of the dermal–epidermal junction, which is consistent with keratinocyte migration and MMP-2 expression. Protease activity is essential for cutaneous wound healing, as demonstrated by the retarded wound healing seen in mice deficient in plasminogen, the serine protease precursor, and mice treated with a metalloprotease inhibitor. Neutrophil elastase has also demonstrated antimicrobial properties against a range of wound-associated bacteria. It is when the balance between ECM degradation and deposition is disrupted, however, in part, due to a disruption in the equilibrium of the production or activation of proteases and their respective inhibitors that wounds become chronic.

Although chronic wounds have long been recognized as a type of inflammatory disease, it was not until the mid to late nineties that a recognition of the relative roles of cytokines and proteases may play in stalled healing [9,10]. Analogously, during that period, burn wounds were characterized for degradation of fibronectin by elastase [11]. Furthermore, early work clearly uncovered the mechanism resulting from high concentrations of proteases in wound exudate to degradation of key growth factors and extracellular matrix proteins that mediate cell proliferation and tissue structure and observed in a stalled inflammatory stage. Both host and bacterial proteases play a role in prolonging chronic wounds [12]. Thus, a prolonged high concentration of proteases stemming from neutrophils, bacteria, and macrophages results in the breakdown of extracellular matrix formation and growth factor activity necessary to progress into granulation tissue formation.

### 1.3. Chronic Wound Protease Modulating Dressings

In the early 2000s, treatment options for protease elevation in chronic wounds were proposed based on the inhibition of destructive proteases thought to prolong the inflammatory response [13,14,15,16]. Each approach was based on a design that proposed a different mechanism of action with protease-dressing interactions at a molecular level through an inhibitor, enzyme-dressing salt bridge sequestration, silver nanoparticle protease inhibition, and collagen-substrate uptake. There ensued a number of proposed approaches to protease control, which have recently been reviewed in historical order in a comprehensive report on dressing design and mechanism of action to the issue of modulating protease levels and the current thinking on using this approach in chronic wound care [17].

One approach used as a model on the application of protease inhibitors to lower pathologic levels of proteases in the chronic wounds was based on a cotton-formulated serine protease inhibitor [14]. Subsequently, the mechanism of protease inhibition applicable to chronic wound treatment was extended to include non-toxic inhibitors of Human Neutrophil Elastase (HNE). An interesting example can be found with the elastase inhibitor oleic acid, for which a mechanism of action was delineated based on albumin as an inhibitor transfer-carrier protein [18]. Albumin, which is the most abundant protein in wounds, has been characterized as having sixty-four oleic acid storage binding sites. It was further elucidated that oleic acid’s application to acute wounds accelerates healing [19]. The application of oleic acid and other fatty acids is now an approved treatment for chronic wound patients in some parts of the world [20]. It is interesting that oleic acid’s activity aligns with folklore remedies where olive oil is attributed with wound healing properties.

### 1.4. Hydrogen Peroxide and Wound Healing

Reactive oxygen species perform receptor signaling and act as regulators of signal transduction and gene expression processes [21]. Moreover, in wound repair, ROS are expressed by phagocytic and non-phagocytic cells [22,23]. Hydrogen peroxide levels may give rise to cell proliferation or promote antimicrobial activity, and the mechanism and relative therapeutic efficacy have been addressed in recent studies [22,24,25,26]. Early on interest in therapeutic levels generated by dressings at low-level hydrogen peroxide levels (5–50 µM) have been shown to promote cell proliferation since Schmidt et al. [27] first demonstrated low-level hydrogen peroxide based on compositional functionality in semi-occlusive dressings.

In recent years, hydrogen peroxide has been studied for application to wound infection and promotion of cell proliferation employing different types of electrochemical and bioelectric technologies combined with hydrogels. Moreover, recently, approaches to modulate hydrogen peroxide (H_2_O_2_) levels with bio-electric dressings have been reported [28,29,30]. In addition, the use of glucose responsive, biomimetics, enzymes, and transition metal complex designs, along with injectable hydrogels incorporated into the wound dressing material promote the Fenton reaction at levels that are either antibacterial or promote cell proliferation through hydrogen peroxide-mediated signal transduction.

### 1.5. ESKAPE Pathogens and Wound Dressings

ESKAPE pathogens (*Enterococcus faecium*, *Staphylococcus aureus*, *Klebsiella pneumoniae*, *Acinetobacter baumannii*, *Pseudomonas aeruginosa*, and *Enterobacter species*) have been identified as critical multidrug-resistant bacteria for which effective therapies are rapidly needed [31] and a high priority of the World Health Organization. There are to date no reports of viable hemostatic dressings or disinfectant wipes with robust broad spectrum antibacterial activity against the priority virulent ESKAPE pathogens. To date, there are no reports of viable hemostatic dressings or disinfectant wipes with robust broad spectrum antibacterial activity against the priority virulent ESKAPE pathogens. We have recently reported on the development of dressings with combined hemostatic and antibacterial activity [32].

Based on recent findings the formulation of low levels of ascorbic acid in greige-cotton-containing nonwovens promoted robust antibacterial effect (>99.99% against Gram-positive and negative bacteria as measured by AATCC 100 Test Method) and prompted the development of a dressing with combined hemostatic and antibacterial activity [32]. The efficacy of the ascorbic acid formulated cotton is thought to have as its mechanism of action the generation of antibacterial levels of hydrogen peroxide through reaction with small amounts of copper found in the cotton fiber cuticle. This is a well-characterized mechanism for hydrogen peroxide based on copper redox-catalyzed reactions [33]. Currently, we have determined that ascorbic acid in the presence of the zeolite-treated TACGauze^TM^ improves hemorrhage control comparable to the current standard of field care. Here, in vitro antimicrobial efficacy studies evaluate the efficacy of cotton-based dressings to inhibit the formation of pathogenic bioburden against common and specific ESKAPE wound pathogens.

Here, we discuss the conferred functionalities, including protease modulation, hydrogen peroxide generation, or bacteriostatic activity, review some of the background on relevant wound healing concepts related to chronic wounds, and consider dressing designs that take these three properties into consideration. This is contrasted with recent results from a new cotton-based technology that demonstrates protease uptake, and hydrogen peroxide generation and activity against ESKAPE pathogens.

## 2. Results

### 2.1. Protease Modulation

**Figure 2:**
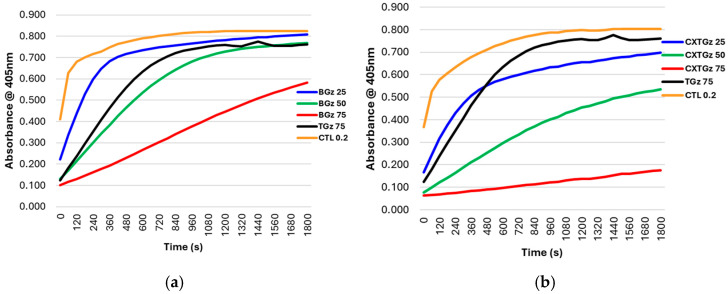

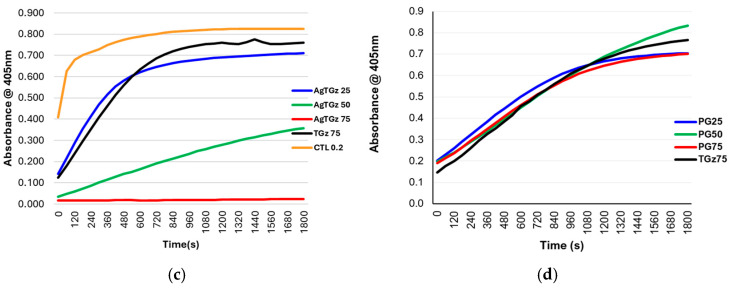
Graphs depict reaction progress curves of the TACGauze (TGz)-treated fabrics (**a**) BIOGauze(BGz), (**b**) Citrate gauze (CXTGz), (**c**) Silver gauze (AgTGz) and (**d**) commercial bandage Promogran (PG) recovered solutions of elastase, 0.2 units/mL for gauze and 0.1 units/mL for PG, after soaking in it for 2 h. Varied weights of samples (25 mg, 50 mg, and 75 mg) were compared with untreated TGz, 75 mg. Substrate hydrolysis was performed with MeO-Suc-Ala-Ala-Pro-Val-pNA, and absorbance monitored at 405 nm, 37 °C. Data are the average of triplicate determinations.

**Figure 3:**
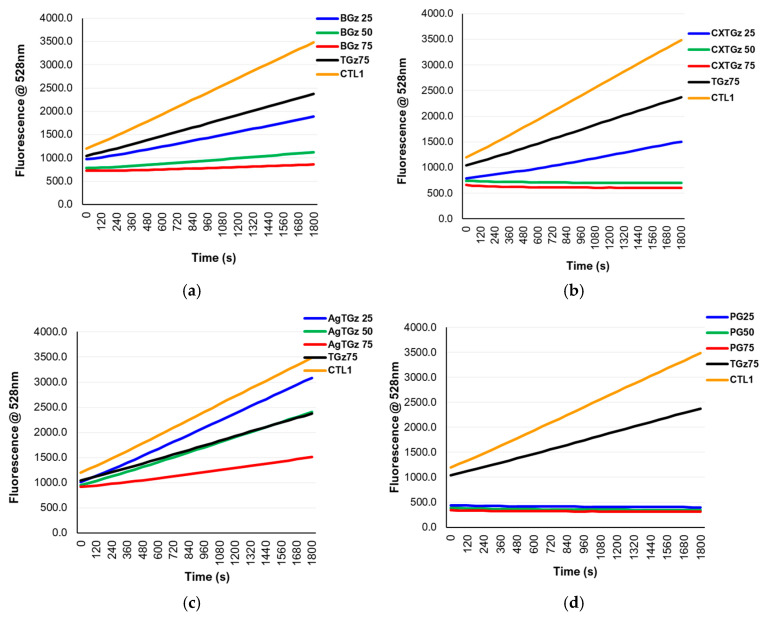
Graphs depict reaction progress curves of TACGauze (TGz) treated fabrics (**a**) BIOGauze (BGz), (**b**) Citrate gauze (CXTGz), (**c**) Silver gauze (AgTGz) and (**d**) commercial bandage Promogran (PG). Recovered solutions of collagenase, and 1 unit/mL, after soaking in it for 2 h. Varied weights of samples, 25 mg, 50 mg, 75 mg, were compared with untreated TGz, 75 mg. Substrate hydrolysis was performed with dye quenched (DQ™) gelatin, fluorescein conjugate, and fluorescent emission monitored at 528 nm. Data are the average of triplicate determinations.

This section compares commercial dressings with protease-modulating activity with a group of cotton-containing dressings having protease-lowering activity and a well-characterized protease-modulating dressing, oxidized regenerated cellulose (ORC)/collagen matrix. We employed hemostatic dressing TACGauze (TGz), which has recently been characterized as a cotton-based technology applicable to acute wounds [34]. We created analogs of TGz modifying with ascorbic acid BIOgauze (BGz), a citrate-ascorbate covalently bonded analog Citrate gauze (CxTGz), and an embedded TGz with nanocrystalline silver (AgTGz), which confers antibacterial activity. These dressing modifications were compared with a commercial dressing, which is described in detail below as a protease-modulating dressing: Promogran, an ORC–collagen matrix. An assessment of the ability of these dressings to bind both HNE and collagenase was made. The results are shown in the reaction progress curves for lowering protease activity in Figure 2 and Figure 3.

The three modified cotton-based dressings (BGz, CxTGz, and AgTGz), which are compared with the TGz and ORC parent dressings for protease-lowering activity demonstrated different levels of dose response based on the effect of dressing mass on protease-lowering as measured with enzyme reaction progress curves that reflect rate of substrate turnover and are designed as a model to reflect the protease activity analogous to dressing treatments and uptake of proteases from chronic wounds. Thus, a higher protease-lowering effect corresponds to less enzyme-substrate turnover as reflected by a reduction in the enzyme-substrate reaction progress curve. For example, BGz showed a step-wise decrease in both elastase and collagenase activity with protease-lowering activity corresponding to a decrease in the reaction progress curve relative to control in the order of decreasing dressing mass (75 mg > 50 mg > 25 mg); less enzyme-substrate turnover at 75 mg followed by the treatments at 50 mg and then 25 mg, which resulted in the most rapid substrate turnover since less enzyme is removed by the dressing and more protease remains in solution. 

On the other hand, CxTGz protease-lowering ability showed a similar dose response curve for elastase. However, the response of collagenase to CxTGz was complete removal of activity from the solution at 50 and 75 milligrams. This may be due to the binding of negatively charged ascorbate and citrate-ascorbate, which are either formulated or covalently attached to cellulose in the dressings, and the citrate-ascorbate would be expected to bind zinc (a factor in collagenase) [35]. The effect of having nanocrystalline silver impregnated in the dressing (AgTGz) showed a complete reduction in elastase activity at 75 mg and a sequential decrease in enzyme activity from 50 to 25 milligrams. Collagenase response to AgTGz was 75 mg > 50 mg > 25 mg. ORC/collagen matrix (Promogran) completely lowers collagenase activity as would be expected since the dressing is a collagen-based material, and this is consistent with the binding of MMPs to ORC. However, it was observed that less ORC matrix dressing mass potentiated the removal of elastase activity, which may be due to the swelling of the dressing in vitro and prevent mass action protease binding the dressing.

To compare with these results, it is important to consider another approach reported [17,36] with a protease sequestrant dressing based on modified cotton textile fibers designed to bind and neutralize the destructive activity of HNE and Matrix Metalloprotease (MMP) in chronic wound pathology [15]. One form was phosphorylated cotton with United States Food and Drug Administration (FDA) approval introduced in the early 2000s [37].

**Figure 4:**
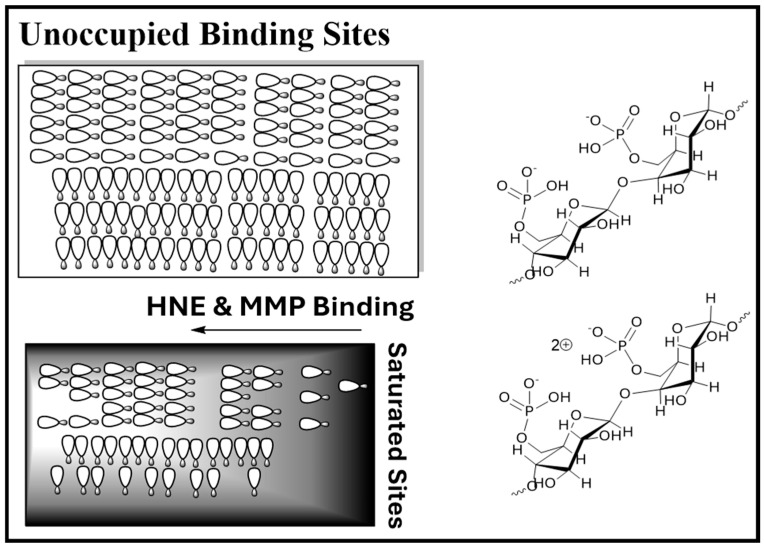
Depiction of the mode of action of protease-lowering activity of phosphorylated cellulose in cotton dressing designed for chronic wounds. In the case of HNE, phosphate groups bind to positively charge arginine side chains. In the case of MMP, phosphate groups bind to zinc. The molecular model depicts binding of HNE to a derivatized cellulose analog at enzymes the active site as another mode of action.

Subsequently, an approach reported by Vachon and Yager employed a sulfonated polymer [38]. The mechanism of action is thought to be based on binding the cationic portion of serine proteases and the zinc-binding site of metalloproteases (Figure 4). As shown by Tarlton et al., the binding of metal ions may also play a role in protease-lowering activity [39]. The intended commercialization of the phosphorylated cotton dressing was to provide a low-cost Medicare reimbursable dressing for chronic wound treatment [37]. Noteworthy as well, since phosphate binds to the mannose-6-phosphate receptor of macrophages it is thought that this would allow some improvement in macrophage up take and growth factor release [40]. A notable example of a polysaccharide-based approach to wound healing that is resistant to protease is the success of Beta-glucans toward stimulating macrophages, fibroblasts, and keratinocyte proliferation [41]. A variety of approaches to protease modulation have been proposed and commercial dressings developed for chronic wound treatment [42,43,44].

### 2.2. Composite Matrix Dressing

The results include a commercial dressing Promogran that as a protease-modulating dressings is a design that has been examined extensively for both clinical efficacy and mechanism of action. The dressing combines oxidized regenerated cellulose (ORC) and collagen (ORC/collagen) matrix. It is important to note that the use of collagen is consistent with promoting protease modulation since elevated MMP in chronic wounds degrades collagen and inhibits incorporation in the ECM. With the idea of promoting granulation tissue formation, it was initially shown in an ex vivo diabetic ulcer model that an ORC matrix reduced elevated chronic wound proteases including elastase, plasmin and matrix metalloprotease [13]. In a comparative study of dressing types composed of collagen, cellulose, and ORC, an analysis contrasting the relative roles of collagen, cellulose, ORC, and ORC/collagen to lower elastase activity was made. Thus, the relative role of charge versus collagen as a substrate sponge is an important consideration to elucidate the dressing’s mechanism of action [45]. An earlier, 33-patient study that examined growth factors, proteases, and protease/inhibitor ratios documented a significant reduction in MMP-9/TIMP-2 ratio ORC/collagen [46]. The results of these studies gave credence to a report on the successful use of ORC matrix in hydroxyurea-induced leg ulcers that can occur with patients being treated for myelodysplastic cancer disorders [47]. Subsequently, years later, a parallel study compared the use of ORC with and without autologous growth factors in patients with diabetic foot ulcers: the authors concluded that the protease-modulating dressing acted synergistically with the growth factors and enhanced their efficacy in reducing the dimensions of the ulcers [48]. 

The correlation of lowered elastase and plasmin activity in chronic wound pressure ulcers with clinical observations on faster healing rates was reported in subsequent years [49]. Notably, two other reports correlated a reduction in protease levels in chronic wounds with ORC/collagen treatment. For example, Smeet et al. demonstrated a reduction in elastase and plasmin in patients in chronic venous ulceration, and Ulrich et al. reported reductions in elastase, plasmin, and gelatinase [50,51]. In recent years, a systematic review and meta-analysis that compiled clinical and mechanism of action reports of ORC/collagen dressings in twenty different published studies indicated a statistically significant effect for wound closure (*p* = 0.027) with the overall effect being 3.4 greater wound closure response than what was observed with control dressings, as well as a significant percent wound area reduction (*p* = 0.006).

### 2.3. Hydrogen Peroxide

In an analogous manner, it is important to understand the current depth of applied work on innovations in hydrogen peroxide(H_2_O_2_) technology. In recent years, hydrogen peroxide(H_2_O_2_) has been studied for its application to wound infection and promotion of cell proliferation employing different types of electrochemical and bioelectric technologies combined with hydrogels. Moreover, approaches to modulate H_2_O_2_ levels with bio-electric dressings have been recently reported [28,29,30]. In addition, the use of glucose-responsive, biomimetics, enzymes, and transition metal complex designs, along with injectable hydrogels incorporated into the wound dressing material, promote the Fenton reaction at levels that are either antibacterial or promote cell proliferation through hydrogen peroxide-mediated signal transduction.

We have endeavored to understand the modification of the unbleached cotton fiber to produce sustained levels of hydrogen peroxide. It has recently been determined that greige cotton generates low levels of H_2_O_2_ (5–50 micromolar). However, the mechanism of action of hydrogen peroxide production in the cotton fiber may be considered multifactorial, i.e., trace metals [52,53], polyphenols [52,54], peroxidase [54], super oxidase dismutase [55,56], and pectin [54,57], which are found in both brown and white cotton varieties that have been correlated with activity [52]. Here, we contrast a new cotton dressing technology that gives hydrogen peroxide activity with a series of dressings from different categories of occlusive chronic wound dressing applications.

**Figure 5:**
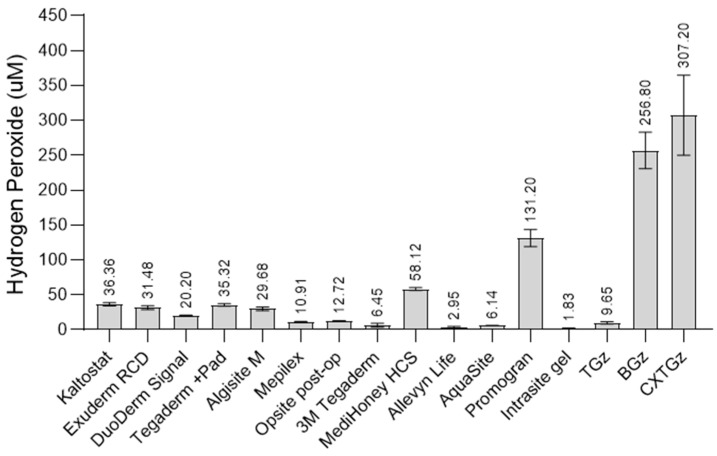
Bar graph displays the amount of hydrogen peroxide (H_2_O_2_) generated in 24 h, determined with the Amplex Red Assay by different representative types of commercial dressings, including TACGauze (TGz), and treated TGz nonwovens, BIOGauze (BGz), and citrate crosslinked (CXTGz).

As shown in Figure 5, hydrogen peroxide generation was observed in different representative types of dressings, including films, foams, hydrogels, composites, and alginates, defined in Table 1. For example, hydrogen peroxide levels varied within a wide range of concentrations associated with levels that have previously been shown to give either to cell proliferation promoting or antibacterial. The order of hydrogen peroxide generation from high to low concentrations was observed to be in the order of composite (collagen + regenerated cellulose) > hydrocolloid (varied types: honey, alginate or carboxymethylcellulose + gelatin) > film > foam > hydrogel. Most of the hydrogen peroxide concentration observed fell within the range documented for stimulating cell proliferation. However, it is important to note that the concentrations reported in this study would not necessarily reflect those accessible inter-cellularly within a wound.

Hydrogen peroxide levels from TGz were within the lower range associated with cell proliferation, whereas BGz and CxTGz gave rise to levels associated with antibacterial activity. This is consistent with an endeavor to understand modification of the unbleached cotton fiber to produce sustained levels of hydrogen peroxide; it has recently been determined that greige cotton generates low levels of H_2_O_2_ (5–50 micromolar). In the case of these dressing analogs, we have previously shown the connection between hydrogen peroxide generation and antibacterial activity. However, the mechanism of action of hydrogen peroxide production in the cotton fiber may be considered multifactorial, i.e., trace metals [52,53], polyphenols [52,54], peroxidase [54], super oxidase dismutase [55,56], and pectin [54,57], which are found in both brown and white cotton varieties that have been correlated with activity [52].

Several reports on the use of electrochemical bandages that deliver monitored levels of hydrogen peroxide have shown promise by inhibiting pathogen growth while not changing the host response. For example, MRSA pathogens were inhibited using this type of approach employing wound-based monitoring of ROS levels [58,59], whereas Banerjee et al. reported improved keratinocyte migration through the generation of an electric field from the bioelectric dressing [60]. Similarly, multifunctional sol–gel hydrogels that employ the incorporation of organometallic complexes linked to glucose oxidase in the hydrogel matrix degrade excess blood glucose to hydrogen peroxide and gluconic acid with an accompanying colorimetric response. These were reported both for antibacterial and photothermal activity with a molecular and cellular explanation of the underlying mechanism of action to accelerated healing [61,62]. Glucose and pH-responsive hydrogels and films have also been reported.

The application of hydrogen peroxide as an antibacterial has been known for over a century, yet it has been re-visited only recently in the context of biofilms [63]. Notably, the recent interest in the use of honey to treat wounds may be partly the result of its production of low levels of hydrogen peroxide [64,65]. Notably, the differences in wound healing upon application of the two functional levels of H_2_O_2_ have been characterized in an in vivo model [22], and characterized based on clinical dosage for the potential to use an economical and naturally occurring material sources for a chronic wound dressing [66]. Reports on the positive efficacy of H_2_O_2_ delivery in vivo merit the development of such promising materials [22].

### 2.4. Antimicrobial Activity of Treated TGz: “ESKAPE” Model

The design and proposed basic mechanisms of the BGz, CxTGz, and AgTGz have recently been reported [32]. Here, we show the antibacterial effect on ESKAPE pathogens in Table 2. It is interesting that TGz demonstrated a significant level of antibacterial activity against all ESKAPE pathogens ranging from 98.44 (1.8 log reduction) to 99.17 (2.1 log reduction). However, it was five logs less than the modified forms. On the other hand, CxTGz was >99.99 percent antibacterial against all ESKAPE pathogens and BGz similarly except for *P. aeruginosa* (15,442) (99.99). Thus, since the mechanism of action is thought to be the generation of hydrogen peroxide, the higher levels of hydrogen peroxide associated with CxTGz may be responsible for the higher antibacterial efficacy against *P. aeruginosa*. AgTGz demonstrated >99.99 against *S. aureus* and *K. pneumoniae*, 99.99 against *A*. *baumannii* and *P. aeruginosa*, and 99.92 and 99.95 against *E. cloacae* and *E. Faecium*, respectively, with a log reduction of 3.1 to 3.9.

It is helpful to underscore the relevance of these results in light of recent reports on the epidemiology and mechanisms of action of ESKAPE microorganisms [31]. To date, ESKAPE pathogen virulence and the widespread effect on the world population, as characterized by the World Health Organization (WHO), have been recently reviewed [68]. Considering this, the results shown here demonstrate some promising potential applications of the modified forms of cotton-based fiber technology for wound dressings and healthcare barrier disinfectants but will require more rigorous testing in the form of surface contact and biological milieu challenges. ESKAPE pathogens remain most challenging in healthcare and battlefield environments where nosocomial and prolonged field care (PFC) exposure infections remain untreated and become critical and associated with high mortality. For example, some of the ESKAPE organisms such as *S. aureus* pose a threat both clinically and with PFC. Moreover, others are found to produce a considerable risk to long-term and critically ill patients, and under certain surgical or urinary tract procedures that have been documented with medical devices. Some remain a threat to the elderly and immunocompromised. The critical issues are prevention and treatment; prevention to stem the threat of acquiring ESKAPE pathogen-based infections in which treatment becomes more challenging. For example, some studies characterize virulence under some clinical settings in terms of 30-day mortality. Thus, the importance of prevention though improved barriers to infection considering the widespread resistance to treatment over the last three decades, new forms of treatment as with boronic acid have recently been introduced with some promise.

Here, all the hydrogen peroxide-generating cotton-based fabrics demonstrated antibacterial efficacy at levels of >99.99 percent except for BGz against *P. aeruginosa* at 99.99 percent though is within the range acceptable by the FDA. This too considers no apparent neutralization of activity by catalase as would be found with *S. aureus*. However, we have not examined hydrogen peroxide resistance in this study. Moreover, it has been previously noted that hydrogen peroxide resistance [69] is inducible in certain bacteria following non-lethal exposure.

**Figure 6:**
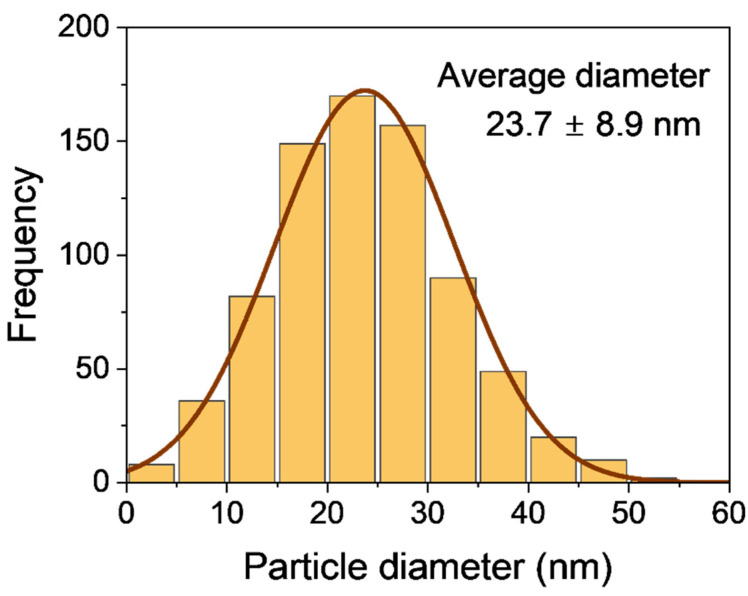
Histogram for the diameter distribution of silver nanoparticles synthesized using raw cotton fibers. The solid line represents a Gaussian fit to the size distribution, with an average diameter of 23.7 ± 8.9 nm.

The lower efficacy of the nanocrystalline silver analogs needs to be revisited bearing in mind levels of nanocrystalline silver in the cotton influence antibacterial activity. Nam et al. showed that at a nanocrystalline silver concentration of 3017 mg/kg reduction of bacteria at 99.998% and 99.994% for *P. aeruginosa* and *S. aureus*, respectively, when prepared on cotton fabrics [70]. On the other hand, for raw white cotton fibers, which produced silver nanoparticles at a concentration of approximately 2100 mg/kg, reductions of 99.99% for *K. pneumoniae* and for *S. aureus* were achieved [71,72]. In this study, the concentration of silver nanoparticles in the fabric AgTGz was much less at 215 mg/kg. In Figure 6, the size distribution curve of the silver nanoparticles in the fabric surface can be seen. As Wright et al. reported as early as the 2000s, dressings containing silver nanoparticles have been shown to be effective in protease modulation [16]. Recently, silver nanoparticles have been incorporated into carrageenan, an Acticoat dressing, and have been shown to be effective in protease modulation [73]. Thus, it is thought that these levels reported here can be improved and performance enhanced.

## 3. Discussion

We examine in this paper the role of wound dressings in modulation of proteases, hydrogen peroxide generation, and concomitant antibacterial efficacy through functionality designed to improve the landscape of wound healing. This approach is one that putatively goes hand in hand with wound care diagnostics [74]. However, though numerous efforts have been made over the last thirty years to integrate therapies with point of care diagnostics, there is absent an overarching integrated approach that brings physical and molecular markers to bear in one therapeutic space. Coordinated approaches are assumed to be part of visual assessments based on TIME (tissue, infection moisture and edge) and have advanced wound healing outcomes as the goal [75]. However, despite these advances, integrating diagnosis and treatment and developing guidelines based on a measurable marker and smart dressing technology have been hampered due to development and treatment costs, biomaterial proof of purpose, updating uniformed guidelines and clinical and regulatory issues in the maze traveled from innovation to successful wound care breakthroughs. Moreover, the variation and complexity of wound pathology is challenging, and though progress has been made toward developing mechanism-based dressings there are to date no ideal dressings or monitoring approaches that comprehensively address marker involvement in chronic and burn wounds. 

It is important to recognize that a mechanistic approach to wound healing was spawned in the early sixties when Winter [76] and Hinman [77] showed that the rate of re-epithelialization increases in a moist wound versus a wound kept dry [76,77]. Collagen at the interface of the scab and dermis impedes epidermal cell movement. The proper regulation of water vapor from a wound to the atmosphere promotes a moist microenvironment allowing epidermal barrier function to be rapidly restored. The concept prompted a revolution during the 1970s in the production of new types of wound dressings to address wound exudate volume regulation versus moisture. Thus, the term intelligent dressing was coined based on the introduction of a polyurethane dressing material that gave a self-adjusting water vapor transmission rate (WVTR) [78]. More recently, Xu et al. identified an optimal WVTR for enhanced healing based on associated healing markers [79].

More recently, molecular-based considerations of the proteolytic environment of the chronic wound have been utilized to effect drug delivery, develop clinical ‘sensing assessments, and multimodal hybrid technology that lowers cytokine levels in vivo [80,81,82]. The application of advanced dressing designs to regulate proteases and cytokines has been examined to a limited degree. It is with this in mind that we also are compelled to review dressings that have been reported to have clinical efficacy in this regard. Thus, these dressing motifs may be contrasted with some of the designs from this study in the context of both traditional and recent innovations.

### 3.1. Clinically Relevant Studies on Other Types of Protease Modulation Dressings: Films, Foams and Hydrogels

Protease modulation dressings have been explored over the last thirty years in an all-inclusive spectrum of semi-occlusive dressing categories applied to chronic and burn wounds. Thus, it is important to underline some relevant dressing types that modulate WVTR while having a mechanism of efficacy in wound healing via proteas modulation. For example, films are often employed as a protective layer to facilitate a type of artificial epidermis as seen with Integra dermal regeneration template; a two-layer dressing composed of an outer silicone-based film and an inner layer of crosslinked fibers including collagen. Film dressings have been shown to promote regeneration of the dermal layer. Moreover, the relative role of its interaction with proteases (MMP1 and MMP9) has been examined in pediatric burn wound patients [83,84]. In this study, it was shown that MMP-2 putatively acted as an anti-inflammatory based on the correlation of increased plasma levels peaking at day seven versus decreased MMP-9 levels (a pro-inflammatory) and the role it plays in a time course in degradation of the collagen fibers of the dressing as well as modulating scar formation.

In a mechanistic study reported by Tarlton and Munro that compared several classes of dressings with sulfonated copolymers a foam dressing containing a sulfonated polymer demonstrated efficacy through complete absorption of MMP-9 compared with a hydrogel control dressing [39,85]. The authors state that presence of a sulfonated co-polymer in the dressing modulates proteases by both direct absorption of MMPs and depleting metal ion co-factors resulting in elimination of proteases. Thus, as demonstrated by previous studies both HNE and MMP are absorbed onto dressing consisting of anionic polymers and positively charge metal co-factors [38,86].

An assessment of the effect of NPWT on the expression of proteases and cytokines in wound fluid from stage III-IV pressure ulcer patients was based on the use of a reticulated silver-coated, polyurethane foam attached to a vacuum-assisted closure (VAC) system [87]. In this study the authors examined biomarker analytes for wound healing that included TNF-alpha, IL-1Beta, MMP-2, MMP-3, and TIMP-1. Interestingly, there was a correlation of TNF-alpha and IL-1 with MMP-2 or MMP-3 levels. Although the authors also addressed levels of TIMP-1 and TIMP-1/MMP ratios, the seven-day study demonstrated no detectable differences. In a study on modulation of the burn wound environment, samples from NPWT were compared with blister fluid a nanocrystalline silver and foam dressing in combination with layers of gauze were employed at the wound interface. Among the six proteins found in greater abundance was MMP9, which has been associated with clinical severity. The authors noted that the combination of these dressings at the wound interface may have affected the extraction.

Among hydrogel dressings that are well studied for protease-modulating activity is an analog based on a polyacrylate (PA) sheet hydrogel. The design of the polyacrylate polymer is a high-density ion-charged superabsorbent polyacrylate polymer like those employed in diapers and hygiene products. The authors elucidated two mechanisms of action based on the proclivity of the PA to both remove from wound fluid the divalent ions as calcium and zinc that modulate and serve as cofactors to MMP activity as well as directly bind MMPs ionically [88,89]. Thus, interestingly, the PA dressing depleted chronic wounds of MMP to a level deemed commensurate with an acute wound. Moreover, this study hypothesized the use of PA in the early therapeutic stages of chronic wound treatment.

Humbert et al. showed in a randomized control a decrease in fibrin and necrotic tissue and an increase in granulation tissue after treatment with a protease-modulating polyacrylate-containing hydrogel compared with an amorphous sheet hydrogel [90]. A recent study on cytokine and protease biomarker levels in venous leg ulcer patients, which were compared with split-thickness donor site wounds, and treatment with a protease-modulating hydrogel dressing was assessed [89]. These demonstrated a correlation of a chronic wound’s rise and fall in elastase and certain types of MMPs within a two-week window following dressing application with later wound healing effects observed in granulation tissue formation and epithelialization at six and ten weeks, respectively. For example, there was a steep decrease in neutrophil elastase and MMP-2 in two weeks following application of the hydro-responsive dressing, and the authors note that better healing outcomes were observed for venous leg ulcer (VLU)s that showed a decrease in MMP-1, MMP-2, and MMP-3 after four weeks. On the other hand, MMP-9 in this study remained almost unchanged throughout the study, in contrast with the previously cited studies, and a MMP-9 cited as a biomarker indicative of severity [91]. Also noted was the gradual decrease in elevated collagen and fibronectin until four weeks.

The complex biomolecular composition of honey affords a variety of mechanism-based avenues for the treatment of chronic wounds. The mode of action has been investigated extensively and covers a wide variety of research and clinical perspectives [92]. For example, it is interesting that a honey-based form of Integra wound dressings has been recommended for superficial to partial thickness wounds. In recent years, a report on the pro- and anti-inflammatory profiles of different concentrations of honey revealed that manuka honey had the effect of decreasing inflammatory components including proteinase 3 [93]. An independent case study that monitored patients with Wound Chek revealed that a form of medical-grade honey decreased protease levels in all patients with different types of chronic wounds [94]. Films are often employed as a protective layer to facilitate a type of artificial epidermis as seen with Integra dermal regeneration template; a two-layer dressing composed of an outer silicone-based film and an inner layer of crosslinked fibers, including collagen. This type of dressing has been shown to promote regeneration of the dermal layer. Moreover, the relative role of its interaction with proteases (MMP-1 and MMP-9) has been examined in pediatric burn wound patients [83,84]. In this study, it was shown that MMP-2 putatively acted as an anti-inflammatory based on the correlation of increased plasma levels peaking at day seven versus decreased MMP-9 levels (a pro-inflammatory) and the role it plays in a time course in degradation of the collagen fibers of the dressing as well as modulating scar formation.

### 3.2. Cotton-Based Dressings with Multiple Functionality

Here, we juxtaposed cotton-based dressings as a natural fiber alternative to synthetic fibers. Moreover, greige cotton has advantages over bleached cotton and is advantageous as a non-adherent, hydrogen peroxide-generating fiber that can be modified as shown in these studies. In addition, the effect of hydrogen peroxide generation by different types of moist wound healing dressings is clearly an area that merits further investigation since optimization of existing dressing used for different chronic and burn wound applications could lead to improved performance. As shown in this study, current approaches to innovative dressing design based on naturally occurring fibers such as greige cotton are advantageous in modified forms as with the ascorbic acid modification to inhibit ESKAPE pathogens.

It is noteworthy that the efficacy of the greige cotton-containing dressings of this study demonstrating the lowering of elastase and collagenase activity is consistent with previous studies on modified cotton dressing, as reviewed here. More recently, commercially available hemostatic dressings consisting of greige cotton have been shown to demonstrate hemorrhage control activity at a level commensurate with battlefield and first-responder trauma treatment [34]. The mode of action of the greige cotton-containing dressing is based on surface hydrophobicity, absorption capacity, and charge [95]. Properties that would putatively provide analogous benefit to chronic wounds have been demonstrated and include high absorption capacity, a negative charge to bind positively charged elastase and zinc-dependent collagenase, improved ease of removal afforded by the hydrophobic surface character, relatively low cost, and hydrogen peroxide generation affording antibacterial activity as discussed in this paper.

#### Hydrogen Peroxide Generation and Protease Modulation

Nonwovens were found to produce an effective level of antibacterial activity of up to 99.99% inhibition. The associated mechanism of action is thought to be the generation of hydrogen peroxide from the formulated fabrics. Thus, the antimicrobial activity of the ascorbic acid and cotton nonwoven formularies of this study is thought to be based on the classically characterized Fenton reaction. The molecular mechanism is well characterized: in the presence of metal ions such as copper or iron, ascorbic acid ((R)-5-[(S)-1,2,-dihydroxyethyl]-3,4-dihydroxyfuran-2(5H)-one) behaves as a pro-oxidant by cooperatively binding metal ions to form an organometallic bivalent complex, metal-dihydroxyfuranone complex (MDC); under aerobic conditions, MDC binds oxygen (O_2_), the core oxygen atoms of hydrogen peroxide, which can then dismutate by way of a protonated reactive oxygen species (ROS) to form hydrogen peroxide as the end product [15]. Moreover, relative to this, it is important to note that a weakly acidic wound environment would promote stability of hydrogen peroxide, but an alkaline environment characteristic of chronic wounds may promote instability. The initiation of this molecular mechanism in the spunlaced fabric is conceivable considering both the levels of hydrogen peroxide demonstrated in the treated fabrics and consistent with the presence of the transition metal ions previously characterized in these types of cotton fabrics [8]. An interesting finding of this work is the relative efficacy of the ascorbic acid nonwoven formulations considering previously published studies that highlight their partial antibacterial efficacy. Moreover, the formulated fabrics function effectively in generating hydrogen peroxide levels commensurate with antimicrobial activity [16] for up to two days. Finally, fabric hand properties that render comfort and resiliency applied to human skin have previously demonstrated the wide spectrum compatibility of spunlaced nonwoven dressings based on greige cotton [96].

Dressing properties that promote hydrogen peroxide release in the wound environment, along with the protease-lowering activities, as treated here through the cotton-based technology, underscore the potential for using spunlaced nonwoven greige cotton fabrics in functional dressings. Moreover, the potential use of these types of dressings for the treatment of stage III and IV pressure ulcers is notable. Their efficacy as a hemorrhage control dressing has been proven, and parallel functionalities related to wound healing and biocompatibility are applicable. The judicious modulation of proteases and hydrogen peroxide at levels that would potentiate an improved trajectory of wound healing in chronic wounds is a goal of wound healing sciences. In this regard, it is also relevant to consider synergy between protease modulation and hydrogen peroxide signaling as an important topic for multifunctional dressings such as the cotton-based technology addressed here.

Protease modulation is a complex process as described above and a trajectory to healing involves its balance with effective cell signaling events relevant to hydrogen peroxide. Thus, synergism between hydrogen peroxide signaling and protease modulation is potentially influenced by several factors that influence hydrogen peroxide stability including antioxidants, half-life and membrane diffusion of hydrogen peroxide, pH and Fe^2+^. It also encompasses understanding the localization and protein-protein interactions of hydrogen peroxide sensors to address questions that remain on the role of hydrogen peroxide signaling comprehensively [97]. Thus, an in-depth assessment of these is crucial to developing more effective wound dressings and optimizing healing processes by leveraging the biochemical dynamics that favor a trajectory to healing.

Longevity and durability of functional effects in cotton-based dressings are influenced by multiple factors, including material composition, environmental conditions, and the nature of the wound. While protease modulation and hydrogen peroxide generation offer significant therapeutic benefits, their sustained efficacy requires careful consideration of material degradation and structural integrity. By optimizing these factors, cotton-based dressings can effectively serve both short-term and long-term wound care needs, ensuring continued healing and protection in diverse clinical scenarios. The performance of the cotton-based technology across diverse wound environments is also important to consider in light of the effect of pH and duration of efficacy, which has previously been evaluated in vitro [37]. However, whereas nonwoven cotton-based technology has been developed to effect hemorrhage control efficacy; as tested in the swine femoral artery models, the effect of high exudate levels that may challenge the absorbent capacity of these types of dressings and maintain a stable healing environment in chronic wounds requires further assessment. Therefore, carefully designed studies are required to scrutinize the behavior of cotton-based technology in conditions with copious exudate. This type of analysis will provide valuable insights into whether these dressings can sustain their effectiveness amidst varying levels of wound moisture and secretion, thus ensuring optimal healing outcomes.

## 4. Materials and Methods

### 4.1. Materials

All general chemicals and fabrics were from existing supply/inventory. Ultrapure water (18 Ω), MilliporeSigma, (Burlington, MA, USA), was used as a solvent for formulations. TACGauze (TGz), based on joint research of USDA and now manufactured by Safeguard Medical (Huntersville, NC, USA), was used. It is a non-woven gauze product incorporating a proprietary blend of bleached cotton/synthetic fibers and greige cotton, TRUE COTTON^™^ from T.J. Beall Company(Drew, MS, USA). The treatments of the TACGauze derivatives, BIOGauze, citrate gauze and the creation of blended cotton gauze embedded with silver nanoparticles “SilverTAC” (AgTGz) were published previously [32]. The table of descriptions is included in the Supplementary Information (Table S1).

### 4.2. Enzyme Assays

For both assays, untreated TACGauze (TGz) and treated TGz, BIOGauze (BGz), Citrate Gauze (CXTGz), and “SilverTAC” gauze (AgTGz), made at Southern Regional Research Center (SRRC), New Orleans, LA, USA, were cut out to measure 25 mg, 50 mg and 75 mg. The commercial bandage, 3M™ Promogran Prisma Matrix, was also cut out to weigh the same.

#### 4.2.1. Elastase Assay

Each sample amount was placed in a separate well of 24-well plate with 1 mL of buffer (pH 7.6 buffer composed of 0.1 M sodium phosphate, 0.5 M NaCl) containing human neutrophil elastase (HNE), 0.1 Unit/mL or 0.2 U/mL, from Innovative Research, Inc. (Novi, MI, USA). Samples were allowed to incubate for two hours at room temperature, after which they were removed and placed in a 5 mL syringe and pressed to drain unbound buffer and enzyme. The unbound elastase fractions were combined and assayed for activity. For assay, 200 µL total volume, 100 µL of sample or standard were added to microplate (96-well format) and 100 uL of elastase substrate, methoxy n-succinyl-Alanine-Alanine-Proline-Valine-para-nitroanilide (MeO-nSuc-AAPV-pNA), 1 µmol/mL, and diluted with buffer (ratio of 20 µL of substrate and 80 µL of buffer). Absorbance of para-nitroaniline was observed at 405 nm, 37 °C, using an Agilent Biotek Synergy HTX multimode microplate reader (Agilent Technologies, Santa Clara, CA, USA).

#### 4.2.2. Collagenase Assay

Each sample amount was placed in a separate well of 24-well plate with 1 mL of buffer (pH 7.6 buffer composed of 0.05 M Tris-HCl, 0.15 M NaCl, 5 mM CaCl_2_, 0.2 mM NaN_3_) containing 1 unit of collagenase from Clostridium histolyticum Type IV (Thermo Fisher Scientific, Molecular Probes, Eugene, OR, USA, E12055 component D). Samples were allowed to incubate for two hours at room temperature, after which they were removed and placed in a 5 mL syringe and pressed to drain unbound buffer and enzyme. The unbound collagenase fractions were combined and assayed for activity. For assay, 200 µL total volume, 100 µL of sample or standard were added to microplate (96-well format) and 100 µL of collagenase substrate, DQ gelatin from pig skin, fluorescein conjugate (Molecular probes E12055 component A, D-12054) diluted with buffer (ratio of 20 µL of substrate and 80 µL of buffer). The fluorescence intensity of the proteolysis of the substrate was measured at room temperature, with excitation set at 495 nm and emission at 528 nm using an Agilent Biotek Synergy HTX multimode microplate reader (Agilent Technologies, Santa Clara, CA, USA).

### 4.3. Hydrogen Peroxide Assay

Hydrogen peroxide levels generated by the fabrics was determined using the Invitrogen Amplex Red Hydrogen Peroxide/Peroxidase Assay Kit (Molecular Probes Inc., Eugene, OR, USA) per the manufacturer’s instructions. Five-millimeter diameter discs were cut from each fabric type using a No. 149 Arch Punch (C.S. Osborne & Co., Harrison, NJ, USA). For each fabric type, enough discs to weigh ~10 mg was placed in a 2 mL microcentrifuge tube (4–5 discs) and a 20:1 dilution of phosphate-buffered saline (PBS: 0.1 M sodium phosphate, 0.15 M sodium chloride, pH 7.2) was added, e.g., 0.200 mL of PBS to 10 mg of fabric, for a final concentration of 50 mg mL^−1^. The tubes were incubated for 1, 5, 10, and 60 min, and 24 and 48 h with three replicates per fabric type and time point. A 20 mL aliquot from each tube was used for the assay and the hydrogen peroxide concentration was determined using a Qubit 4 fluorometer (Thermo Fischer Scientific Inc., Waltham, MA, USA) at excitation and emission maxima of 571 nm and 585 nm, respectively. The negative controls for each time point consisted of 20 mL aliquots of PBS only with no fabric.

### 4.4. Antibacterial Testing of Fabric

The antibacterial activity of treated and untreated TACGauze were evaluated using a standard, globally recognized, antimicrobial textile test that provides a quantitative measurement degree of activity, the AATCC Test Method (TM) 100: Antibacterial Finishes on Textile Materials: Assessment of (American Association of Textile Chemists and Colorists, Research Triangle Park, NC, USA). AATCC TM 100 was performed by Situ Biosciences LLC (ISO 17025 [98] and ISO 17034 [67] accredited, Wheeling IL 60090, USA). Firstly, cut-out disks of the treated nonwoven gauze strips were challenged with two exposure methods to standard microorganisms, *Staphylococcus aureus* (4352) and *Klebsiella pneumoniae* (6538), separately, by incubating the microorganism inoculum in contact with the test sample for a duration up to 24 h without drying. Following exposure, the inoculated microorganisms were recovered, and the concentration of the organisms was determined, colony-forming units (CFU)/mL. The antimicrobial performance was determined by a comparison of the recovered organisms from the test and control samples at time 0 and at time 24 h, and was reported as a percent value relative to the control sample material, % Reduction, Log10 reduction. Situ Biosciences ISO 17034 [67] Certified Reference Material (CRM) treated and untreated controls were used. The procedure was repeated for each nonstandard microorganism: *Enterococcus faecium* (0968), *Acinetobacter baumannii* (19606), *Pseudomonas aeruginosa* (15442), and *Enterobacter cloacae* (13047).

## 5. Conclusions

This study has reviewed some of the salient features of wound dressing technology that modulate protease levels and generate hydrogen peroxide to improve wound healing outcomes. The study juxtaposes results showing the efficacy of cotton-technology with some potential to perform analogously by way of protease-lowering and the generation of hydrogen peroxide. The demonstration here that cotton-based dressings modified with ascorbate to generate hydrogen peroxide and effectively inhibit ESKAPE pathogens, as determined by the AATCC 100 assessment, should provide impetus for opening pathways to further evaluate this technology for potential use in wound care and barrier protection in nosocomial environments. It is important to note that to achieve successful integration of cotton technology, a multifaceted approach must be considered. Collaboration between researchers, clinicians, and policymakers will be crucial in addressing regulatory challenges and ensuring cost-effectiveness. Additionally, comprehensive training programs should be developed to enhance clinician awareness and proficiency in using the new technology. By fostering an environment that encourages innovation and adaptation, the adoption of cotton-based technology can be accelerated, ultimately improving patient outcomes and expanding the therapeutic arsenal available to healthcare professionals.

## Figures and Tables

**Table 1:** Types of dressing construction and representative commercial example.

Dressing	Example
Film	Tegaderm™, Opsite™ post-op
Foam	Allevyn™, Mepilex™
NPWT ^a^	Prevena™, Pico™
Hydrogel	IntraSite, AquaSite^®^
Hydrocolloid	DuoDERM™ Signal, MediHoney, Exuderm
Alginate	Kaltostat^®^, Algisite-M
Composite/matrix	3M™ Promogran Prisma^®^ Matrix ^b^

^a^ NPWT—Negative Pressure Wound Therapy. ^b^ Promogran Prisma is a matrix wound dressing comprised of oxidized regenerated cellulose (ORC) and collagen at 45:55% ratio, respectively.

**Table 2:** “ESKAPE Model ^a^” Antibacterial Results reported as % reduction after 24 h.

Sample	*E*. *cloacae* (13047)	*S*. *aureus* (6538) ^b^	*K*. *pneumoniae* (4352) ^b^	*A*. *baumannii* (19606)	*P*. *aeruginosa* (15442)	*E*. *Faecium* (0968)
TGz	99.17% 2.1 log_10_ reduct.	94.31% ^c^ 1.2 log_10_ reduct.	98.71% ^c^ 1.9 log_10_ reduct.	98.83 1.9 log_10_ reduct.	99.03 2 log_10_ reduct.	98.44 1.8 log_10_ reduct.
BGz	>99.99% 7.2 log_10_ reduct.	>99.99%, nocr 5.3 log_10_ reduct.	>99.99%, nocr 6.2 log_10_ reduct.	>99.99% 6.6 log_10_ reduct.	99.99% 3.9 log_10_ reduct.	>99.99% 7.3 log_10_ reduct.
CXTGz	>99.99% 7.2 log_10_ reduct.	>99.99%, nocr 5.3 log_10_ reduct.	>99.99%, nocr 6.2 log_10_ reduct.	>99.99% 5.3 log_10_ reduct.	>99.99% 4.9 log_10_ reduct.	>99.99% 7.3 log_10_ reduct.
AgTGz	99.92% 3.1 log_10_ reduct.	>99.99%, nocr 5.3 log_10_ reduct.	>99.99%, nocr 6.2 log_10_ reduct.	99.99% 3.9 log_10_ reduct.	99.99% 3.9 log_10_ reduct.	99.95% 3.3 log_10_ reduct.
CRM treated	99.99 3.9 log_10_ reduct.	>99.99%, nocr 5.3 log_10_ reduct.	>99.99%, nocr 6.2 log_10_ reduct.	>99.99% 5.7 log_10_ reduct.	99.94 3.2 log_10_ reduct.	99.99 4.6 log_10_ reduct.
CRM UT ctrl	1.41 × 10^8^ CFU/sample	1.8 × 10^6^ CFU/sample	1.42 × 10^7^ CFU/sample	2.78 × 10^8^ CFU/sample	2.52 × 10^8^ CFU/sample	1.8 × 10^8^ CFU/sample

^a^ ESKAPE is an acronym comprising the scientific names of six highly virulent and antibiotic-resistant bacterial pathogens including: Enterococcus faecium, Staphylococcus aureus, Klebsiella pneumoniae, Acinetobacter baumannii, Pseudomonas aeruginosa, and Enterobacter spp. Enterobacter cloacae species is used in this study. Antimicrobial results of fabric samples tested according to AATCC Test Method (TM) 100: Antibacterial Finishes on Textile Materials. ^b^ Standard Test Pathogens. ^c^ TACGauze was tested and the values of the CRM untreated and treated controls are CRM—untreated 2.92 × 10^8^ CFU/sample and 3.13 × 10^7^ CFU/sample; CRM treated 99.96, 3.4 log_10_ reduction and 99.52, 2.3 log_10_ reduction for *K. pneumoniae and S. aureus*, respectively. Certified antimicrobial test using ISO 17034 [67] CRM (certified reference material). The tests were conducted by Situ Biosciences. nocr—no organismal counts recovered. CFU—counts.

## Data Availability

The original contributions presented in the study are included in the article; further inquiries can be directed to the corresponding author.

## Supplementary Materials

The following supporting information can be downloaded at: https://www.mdpi.com/article/10.3390/ijms27020610/s1, Table S1. The definition TACGauze™ fabric nomenclature and treatment.
