# Supplementary material for: Advancements in Functional Dressings and a Case for Cotton Fiber Technology: Protease Modulation, Hydrogen Peroxide Generation, and ESKAPE Pathogen Antibacterial Activity
Source: Int J Mol Sci. 2026 Jan 7;27(2):610. doi: 10.3390/ijms27020610 (PMC12841211; doi:10.3390/ijms27020610)
Supplement: Supplementary file 1 [file ijms-27-00610-s001.zip › ijms-3273165-supplementary.pdf]

Advancements in Functional Dressings and a Case for Cotton Fiber Technology: Protease Modulation, Hydrogen Peroxide Generation and ESKAPE Pathogen Antibacterial Activity

J. Vincent Edwards ,* Nicolette T. Prevost, Doug J. Hinchliffe, Sunghyun Nam, and Crista A. Madison

Southern Regional Research Center, Agricultural Research Service, United States Department of Agriculture, New Orleans, LA 70124, USA; vince.edwards@usda.gov(V.E.);nicolette.prevost@usda.gov(N.T.P.);doug.hinchliffe@usda.gov(D.H.); sunghyun.nam@usda.gov (S.N.); crista.madison@usda.gov (C.M.)

*Correspondence: vince.edwards@usda.gov; +1-504-286-4360

Table S1. The definition TACGauze™ fabric nomenclature and treatment

Fabric Name	Abbreviation	Fabric Treatment[1]
TACGauze	TGz	None
BIOGauze	BGz	ascorbic acid on TACGauze applied by pad-dry-cure method
Citrate TACGauze	CXTGz	crosslinking citric acid and ascorbic acid to TACGauze using pad-dry-cure method
Silver "TACGauze"	AgTGz	silver "TACGauze" version manufactured at SRRC using cotton fibers embed with silver nanoparticles[2]

[1] Reference [32] . [2]Silver nanoparticles were introduced into cotton fibers using previously described methods [71]. Silver impregnated cotton fibers were substituted for the bleached cotton component(20%) of TACGauze, creating silver impregnated TACGauze (AgTGz).
